# The medical humanities at United States medical schools: a mixed method analysis of publicly assessable information on 31 schools

**DOI:** 10.1186/s12909-023-04564-y

**Published:** 2023-09-01

**Authors:** Joshua Anil, Phoebe Cunningham, C. Jessica Dine, Amanda Swain, Horace M. DeLisser

**Affiliations:** Academic Programs Office, Perelman School of Medicine, Jordan Medical Education Center, University of Pennsylvania, 6th Floor, Building 421 3400 Civic Center Blvd, Philadelphia, PA 19104-5162 USA

**Keywords:** Medical humanities, Arts and humnaities in medicine, Narrative medicine, Professionalism and humanism

## Abstract

**Introduction:**

There have been increasing efforts to integrate the arts and humanities into medical education, particularly during undergraduate medical education (UME). Previous studies, however, have focused on courses and curricular programming without rigorous characterization of the associated paracurricular environment or infrastructure enabling or facilitating these offerings.

**Methods:**

To assess opportunities for students to engage the arts and humanities during their medical education as well as the institutional resources to support those opportunities, we developed the Humanities and Arts Programming Scale (HARPS): an 18-point scale involving eight sub-domains (Infrastructure, Curricular Opportunities, Extracurricular Engagement, Opportunities for Immersion, Faculty Engagement, Staff Support, Student Groups, and Scholarship). This scale was used to evaluate the top-31 ranked United States medical schools as determined by US News and World Report’s (USWNR) Medical School Research Rankings using information derived from public-facing, online information.

**Results:**

Mean cumulative HARPS score was 11.26, with a median score of 12, a standard deviation of 4.32 and a score range of 3–17. Neither USWNR ranking nor private/public institution status were associated with the cumulative score (p = 0.121, p = 0.739). 52% of institutions surveyed had a humanities-focused center/division with more than 70% of the schools having significant (> 5) faculty engaged in the medical humanities. 65% of schools offered 10 or more paracurricular medical humanities events annually, while 68% of the institutions had more than 5 medical humanities student organizations. While elective, non-credit courses are available, only 3 schools required instruction in the arts and humanities, and comprehensive immersive experiences in the medical humanities were present in only 29% of the schools.

**Conclusions:**

Although there is a significant presence of the medical humanities in UME, there is a need for integration of the arts and humanities into required UME curricula and into immersive pathways for engaging the medical humanities.

**Supplementary Information:**

The online version contains supplementary material available at 10.1186/s12909-023-04564-y.

## Introduction

Physicians are increasingly viewed less favorably by the public, [1, 2] with perceived losses in physician empathy, compassion and caring cited often as factors contributing to a waning of the public’s trust and respect for physicians. In the context of this public dissatisfaction, physicians experience significant rates of professional dissatisfaction,[3] burnout,[4, 5] depression,[5, 6] and suicide [7]. The recent COVID-19 pandemic has further hardened the distrust and suspicions many patients have of physicians [8–10] as well as exacerbated the professional and emotional challenges of physicians with potentially enduring negative effects [11–13]. There is now a growing consensus, supported by emerging data, that the integration of the arts and humanities into the training and experiences of physicians will be an important element in addressing these troubling societal trends and professional challenges [14–20].

This integration of the arts and humanities into medical education to foster the development of physician competence and professionalism is encapsulated in the term, *medical humanities* [15, 16, 21]. It encompasses a variety of art forms including theater, literature, poetry, visual arts and performing arts, as well as disciplines in the humanities such as ethics, history, philosophy, literature and art criticism and theory. The ability of the medical humanities to (i) promote critical affective, cognitive, relational and communication skills, (ii) offer historical and structural contexts and frameworks, (iii) foster teamwork and collaboration, and (iv) enable opportunities for community and connection, provide compelling reasons for the integration of the arts and humanities into medical education [16].

Calls for humanistic practice in medicine date back to the first decades of the 20th century [17]. While not new, over the last several decades content and instruction related to the arts and humanities have been increasingly included in medical school curricula [22–26]. However, in the past 10 years there have been accelerating efforts from several directions to more coherently, comprehensively and rigorously integrate disciplines, experiences and pedagogy from the arts and humanities into medical education, particularly during undergraduate medical education (UME) [15, 16, 20]. In the United States these efforts have most recently coalesced around the Fundamental Role of the Arts and Humanities in Medical Education (FRAHME) initiative of the American Association of Medical Colleges (AAMC) [16]. With a focus across the continuum of both training and practice, the goal of the FRAHME initiative is *“to improve the education, practice, and well-being of physicians through deeper integrative experiences with the arts and humanities.”*[16]

Recommendations from the FRAHME initiative include increased advocacy for the medical humanities, development of competency-based pedagogy, more rigorous evaluations of educational impact, new approaches using the arts and humanities to support learner and practitioner wellness, increased stakeholder collaborations, faculty development and training and enhanced medical education scholarship [16].

A necessary first step in implementing these recommendations, whether on an individual institutional level or as a part of national/collective initiatives is to define the current state of medical humanities offerings and programing as well as the educational environment and infrastructure for enabling and facilitating the engagement of physician learners with the arts and humanities. Such an inventory would be helpful in defining best practices and measures of excellence. In this regard, important work has been done in cataloguing the “ecology” of medical humanities in medical training [22–26]. These studies, however, are limited in that they have focused on courses and curricular programming without rigorous characterization of the associated paracurricular environment or the infrastructure supporting these offerings that foster the learners’ experiences in the medical humanities. In this paper we describe the integration of the art and humanities across eight domains at 31 US medical schools, findings that have implications for expanding the presence of the medical humanities in physician training and experiences.

## Methods

### Data sources

To evaluate the presence of the medical humanities within UME, a systematic review of public-facing information from the top 30 medical schools from the 2022–2023 US News and World Report (USNWR) Best Medical School: Research list [27] was performed. A tie for rank 30 in the list resulted in the evaluation of 31 total schools. A complete listing of these schools can be found in Supporting Information, Appendix 1. All information used in this study was derived from public-facing, web-based sources accessible through a standard internet browser without a need for a password or other credentials, from a “.edu” site associated with an institution, or a link from such a site. Non-Internet sources of information were not used. The review was primarily restricted to the medical schools of individual institutions; however, pertinent information from outside departments or schools within an institution were considered on a case-by-case basis, if they provided documented services or opportunities to medical students. There was no direct or indirect human participation in our study.

### Data extraction

Medical humanities were broadly defined as any discipline outside of the traditional physical sciences (biology, chemistry, physics, mathematics, computer science and statistics) including, history, literature and writing, art, music, philosophy, anthropology, sociology and integrative disciplines such as social medicine and narrative medicine. Ethics offerings were collected for completeness but were not included in the analyses of this study due to their already extensive inclusion into medical school. Two authors (JA, PC) independently reviewed each of the 31 schools and created individual reports that were not accessed by the alternate investigator. With specific attention to UME, and availability and accessibility to medical students, information was gathered on medical humanities curricula, programming, offerings, research, and other opportunities at each institution. Raw data were noted with collection dates and hyperlinks to original source webpages for reproducibility.

### Data analysis

Given the absence of an appropriate instrument, we developed the Humanities and Arts Programming Scale (HARPS) to assess both the opportunities for students to engage with the arts and humanities as a part of their medical education as well as the institutional infrastructure to support those opportunities. We began with a review of the literature to identify areas that would be indicators of institutional excellence in the incorporation of medical humanities in the educational experience of students. This review provided the basis for the development of the HARPS as an 18-point evaluation scale composed of the following eight domains: Infrastructure, Curricular Opportunities, Extracurricular Programing, Opportunities for Immersion, Faculty Engagement, Staff Support, Student Groups, and Scholarship. The definitions of the 8 domains are in Table 1, while Table 2 describes the rubric for scoring. The two data-collecting researchers independently scored all 31 institutions in all eight categories before coming together with all the investigators to compare evaluations and discuss any differences in scoring. Use of hyperlinks and detailed data-recording allowed for reconciliation of all perceived discrepancies to create a final scoring evaluation for each reviewed institution. Descriptive statistics for each school were calculated using Excel (Microsoft, version). Linear regression analyses with USNWR ranking as the dependent variable and x independent variables were performed using Graphpad Prism (version 9.5.0), which was also used to do the statistical analyses, as well as create the frequency distribution and correlational plots. Further details on the development of the HARPS can be found in Supplemental Information, Appendix 2.

**Table 1:**
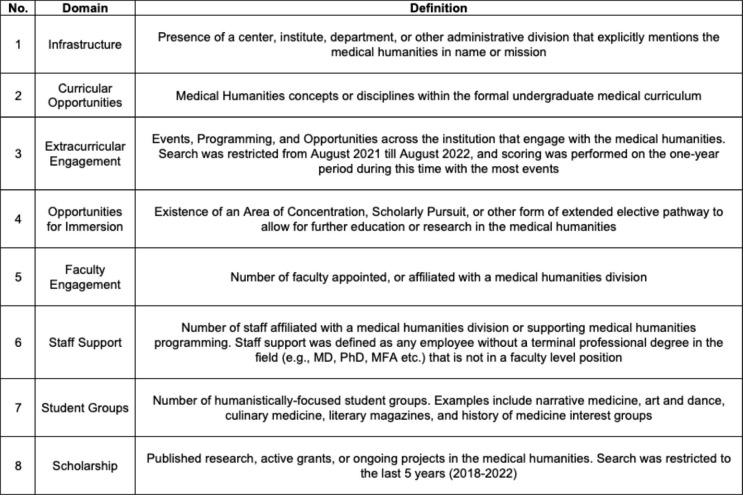
HARPS Evaluation Domains

**Table 2:**
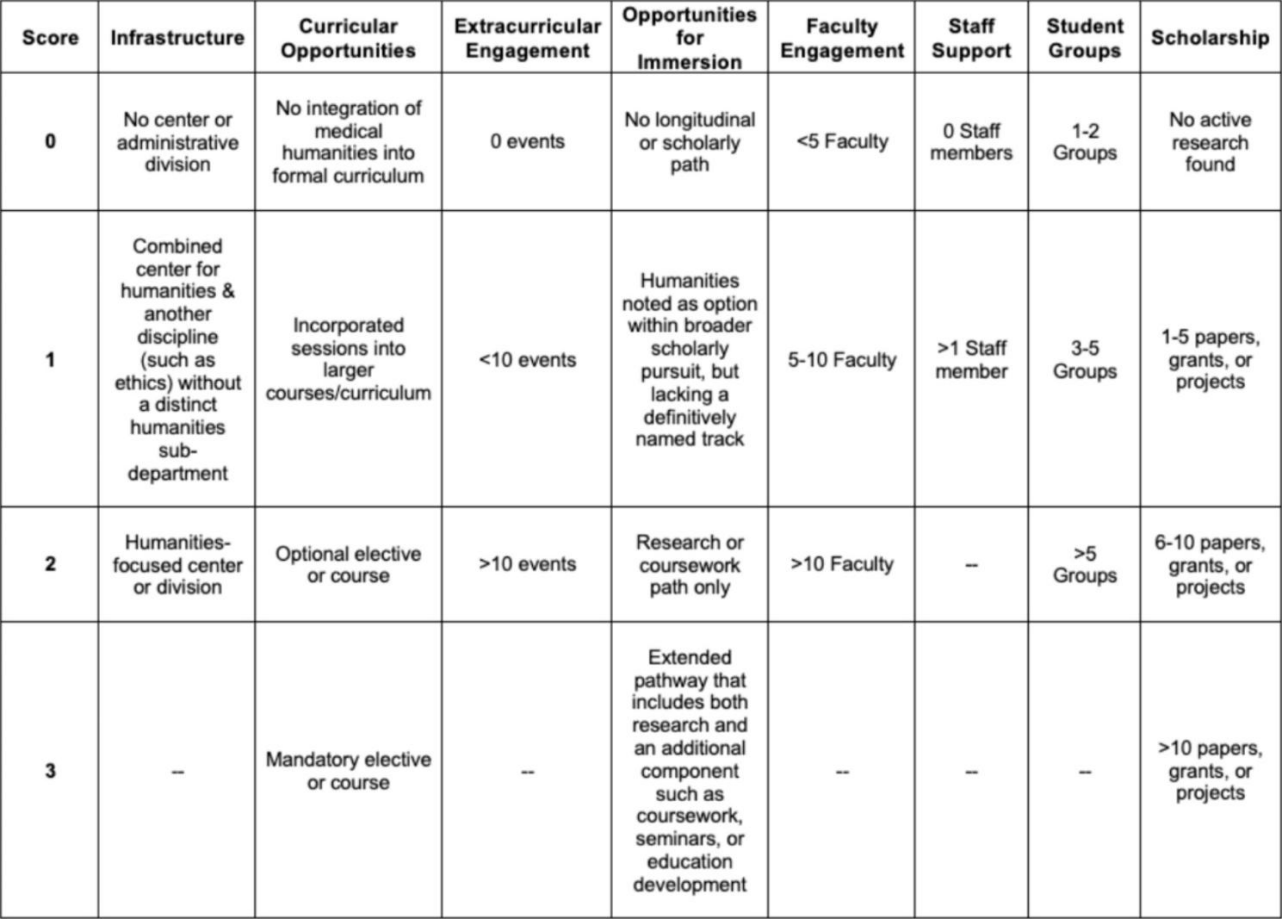
HARPS Scoring Rubric

## Results

### The presence of the medical humanities in US medical schools

Individual domain and cumulative HARPS score data for the group of 31 schools are presented in Table 3. The average total score was 11.26 out of a possible 18 points (62.6%), with a median total score of 12 (66.7%) and a standard deviation of 4.35 (Table 3).

**Table 3:**
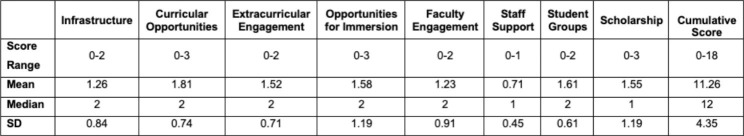
HARPS Scores for the group of 31 medical schools

**Fig. 1:**
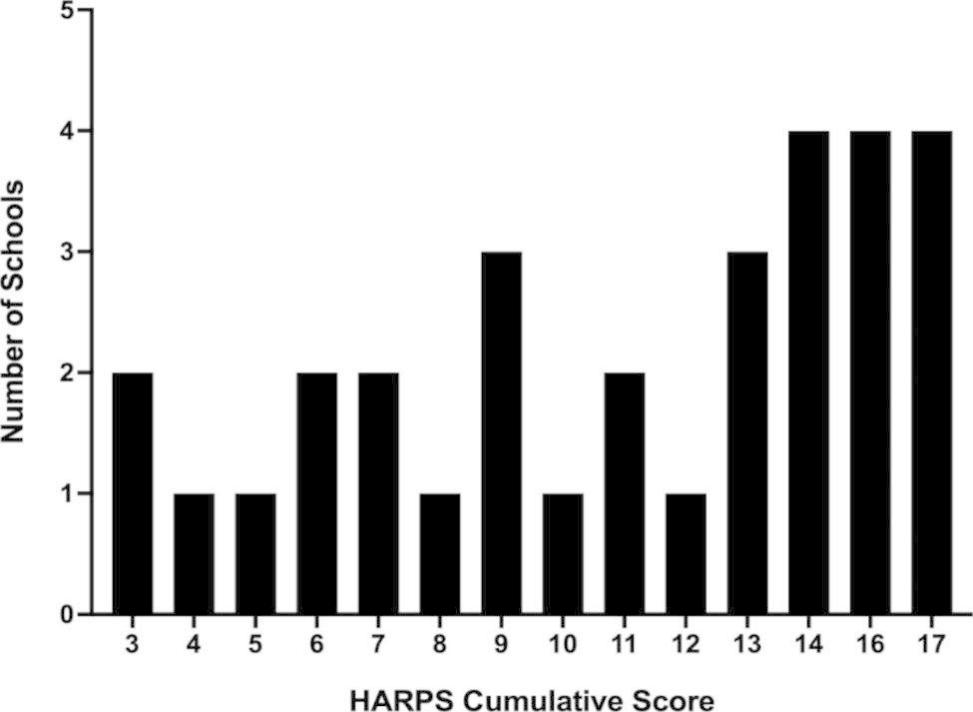
Frequency distribution of the cumulative HARPS score Shown is the frequency distribution the cumulative HARPS score of the 31 schools obtained from summing the scores of the eight individual sub-domains (see Tables 1 and 2). Means, median and standard deviations are presented in Table 3

No institution received a full 18 points, with total scores ranging between 3 and 17 (Fig. 1). Three (9.6%) institutions scored between 0 and 4 points, nine (29.0%) between 5 and 9, eleven (35.5%) between 10 and 14, and eight (25.8%) between 15 and 18. Twenty-three schools (74.2%) received more than 50% of available points (score > 9).

We also assessed whether there was an association between the cumulative HARPS score and the public versus private status of an institution (Table S2, Fig. S1). We found that a school’s status as a public versus a private entity was not significantly associated with the HARPS cumulative score (10.92, SD 3.68 versus 11.47, SD 4.72, p = 0.626, Mann-Whitney Test, two-tailed) (Fig. 3). This remained true even when restricting analysis to the institutions with total HARPS score in the top 75th and top 50th percentile (Chi-Square, p = 0.355, p = 0.379, respectively). Public institutions were among the highest scorers, with two schools scoring in the 15–18 points range. Notably, the lowest scoring institutions were all private, with no public school scoring fewer than six points.

### The medical humanities by domains of analysis in US medical schools

Data (Fig. 4) for each domain of the HARPS along with a pertinent representative institutional example are presented below.

#### Infrastructure: centers, institutes and programs

Sixteen medical schools support centers, institutes, or departments dedicated to the medical humanities or social sciences (Fig. 4A). These schools either had a center focused solely on the medical humanities or a shared center with a clear subsection or division dedicated to the medical humanities. Conversely, seven schools had centers where medical humanities or social sciences were a component of the program. This most frequently included centers that merged medical humanities and bioethics but did not have a distinct subsection or subdepartment dedicated to the medical humanities (Fig. 4A). For eight schools there were no current centers which included the medical humanities or social sciences (Fig. 4A).

Founded in 1999, Trent Center for Bioethics, Humanities and History of Medicine at Duke University School of Medicine [28] has continuously supported interdisciplinary learning, with each of the listed domains - bioethics, humanities, and history of medicine – having distinct programing within the center. These programs include curricular initiatives, scholarship, and creative projects aimed at bringing together scholars throughout the larger community to foster research and other collaborations. Faculty within bioethics and history of medicine also teach courses accessible to undergraduate medical students. Overall, their program emphasizes how medical humanities intersects broadly with domains within and outside medicine and creates spaces to learn and explore these intersections.

#### Curricular opportunities

The majority of schools (22/31, 70.1%) received a score of 2 for curricular engagement, indicating they offered optional (not required for graduation) elective courses in the medical humanities (Fig. 4B). Within this cohort there were a diversity of offerings, with some institutions offering limited panels of 1–2 electives during the preclinical period, while others offer many electives in a variety of disciplines across the pre-and post-clerkship periods. Of the remaining nine schools, three (9.6%), had courses in the arts and humanities as requirements for graduation, three integrated humanistic themes into larger courses and for the remaining three, evidence for a medical humanities curriculum could not be found.

Vagelos College of Physicians and Surgeons at Columbia University provides an example of this mandatory incorporation, as first-year medical students must complete a half-semester seminar in the humanities [29]. The curricula include 12–15 seminars offerings each year, taught either by physicians or by relevant arts and humanities professionals, in areas such as dance, literary studies, visual arts, narrative medicine, history of medicine, photography, film, and religious studies. The variety of disciplines allows students from a diversity of backgrounds to find content that engages them. Further detail on institutional coursework and electives can be found in Supplemental Information Appendix 3.

#### Extracurricular engagement

The majority of schools (20/31, 64.5%) offered 10 or more events across a one-year period, with seven schools offering less than 10 events and four schools offering none (Fig. 4C). Of the schools with events, programming included weekly conversations on arts and humanities, author talks, and performances. Some events were hosted through the schools’ respective medical humanities or social science institutes while others were organized by student groups.

The University of Pittsburgh supports initiatives across medical history, religion and other humanities [30]. The CF Reynold Medical History Society is a Pittsburgh-based medical history and humanities organization that hosts free public lectures. Some lecture examples include *Defining and Treating Heart Disease: A History, What is the Sound of History?* and *The Revolutionaries in the History of Radiology*. The Health Humanities Lecture Series aims to connect issues in disability, embodiment, trauma, with the arts, humanities and sociocultural studies. There are up to 11 lectures each year. The University of Pittsburgh also supports a Healthcare and Religion lecture series, a religion resource map, and a podcast titled *Remains to be Seen*.

#### Opportunities for immersion in the medical humanities

There was a wide distribution of longitudinal, immersive opportunities available to students (Fig. 4D). Nine (29%) medical schools provide a defined track or area of concentration that includes both curricular and research components. Nine (29%) schools provided dedicated research paths. Four medical schools provide opportunities to do research in the medical humanities but not as a defined program. Lastly, for nine medical schools a specific opportunity for an immersive research or curricular experience or pathway in the medical humanities could not be identified.

The Case Western School of Medicine’s Humanities Pathways exemplifies a multifaceted, longitudinal learning opportunity [31]. Each year, 8–10 students are accepted and supported by 20 faculty on the Pathway Advisory Committee. Throughout the first and second years there are weekly seminars averaging to 3–4 h per week. In addition, students take courses at the University in the humanities or social sciences. This foundational knowledge is then translated into a final scholarly project under the guidance of a mentor.

#### Faculty and staff engaged in or supporting the medical humanities

While 18 (58%) of the evaluated institutions had more than 10 faculty members formally affiliated with the medical humanities, a more polarized distribution was seen (Fig. 4E). Nine institutions (29%) had between 0 and 5 faculty, while only four (12.9%) had between 5 and 10 faculty. Despite the variation in faculty engagement, 22 (70.9%) of schools had more than one administrative or staff employee involved with medical humanities programming (Fig. 4F). Notably, not all of the institutions with significant faculty support had dedicated administrative staff. Two programs with 5–10 faculty were found to not have any staff, while three institutions with less than five faculty did have additional staff support. Non-faculty, staff roles ranged from administrative personnel to writers/artists-in-residence.

Stanford University School of Medicine provides an example of how diverse positions and responsibilities can create a robust educational and scholarly environment for medical humanities. Housed within the school’s Biomedical Ethics Program, Stanford’s Medicine and the Muse has a large team drawn from a variety of disciplines and educational levels [32]. The 13-member steering committee contains medical students, residents, attending physicians and several faculty members from humanities and arts departments across the university. This steering committee oversees the work of an Executive Director and Program Director that perform the daily administration of the program. The Medicine and the Muse program also houses a visiting faculty Writer-in-Residence as part of the core team. Outside of this core, 58 additional faculty from both the medical school and the broader university participate as affiliated faculty.

#### Student groups

Student groups were an area of strength across the surveyed institutions (Fig. 4G). Twenty-one (67.7%) of institutions had more than five medical humanities-related organizations. Another eight (25.8%) had three to five groups, with only two schools having fewer than three of these student organizations. The interests of these groups were varied and included narrative writing, culinary medicine, dance, and medical history. Literary and art magazines are common, present at 77.4% of institutions.

Ohio State’s medical school provides an example of the breadth of student-run medical humanities student groups [33]. In addition to their literary and arts magazine, Ether Arts, Ohio State boasts an orchestra, dance club, and an acapella singing group. The school also hosts a writing group, theater/film in medicine organization, and a photography club. Stretching beyond the more common disciplines in the medical humanities, Ohio State students have created an improvisational acting group teaching students how to use improv skills in clinical encounters, a cultural cooking class to build cultural, historical, and social competency, and even a crochet organization that supports local hospitals while instilling a culture of service. Full details on student groups can be found in Supplemental Information Appendix 4.

#### Research/scholarship related to the arts and humanities

Almost a third of medical schools (10/31) noted more than 10 research projects (Fig. 4H) in the past five years. Of the remaining schools with research projects, five (16.1%) and eight (25.8%) respectively noted 6–10 and 1–5 research projects. Eight schools (25.8%) had no identifiable ongoing research projects. Both faculty-led scholarship and student-led projects were identified.

UCLA’s Center for Social Medicine and Humanities places an emphasis on applying these domains to address health, disease, and medicine through the lens of social justice [34]. The faculty’s scholarship highlights the interdisciplinary nature of the center, with faculty possessing backgrounds in medicine, anthropology, and history all represented. With varied expertise, research touches on the history of psychiatry, legacies of imperialism in global HIV/AIDS responses, perceptions of illness, ethnographic studies of physician-patient subjectivity, and interpersonal violence.

## Discussion

Previous studies on the presence of the medical humanities in UME have focused on curricular programing without explicit reference to the associated paracurricular environment or institutional infrastructure that facilitates students’ engagement in the arts and humanities [22–26]. In this paper, we used a novel, evidence-based, multi-domain scale (HARPS) we developed for assessing the opportunity for students at an individual medical school to engage with the arts and humanities as a part of their educational experience. Data derived from this instrument enables us to provide the first-of-its-kind-description of the state of medical humanities in UME, highlighting both areas of progress as well as opportunities for growth that we believe will foster the integration of the arts and humanities.

**Fig. 2:**
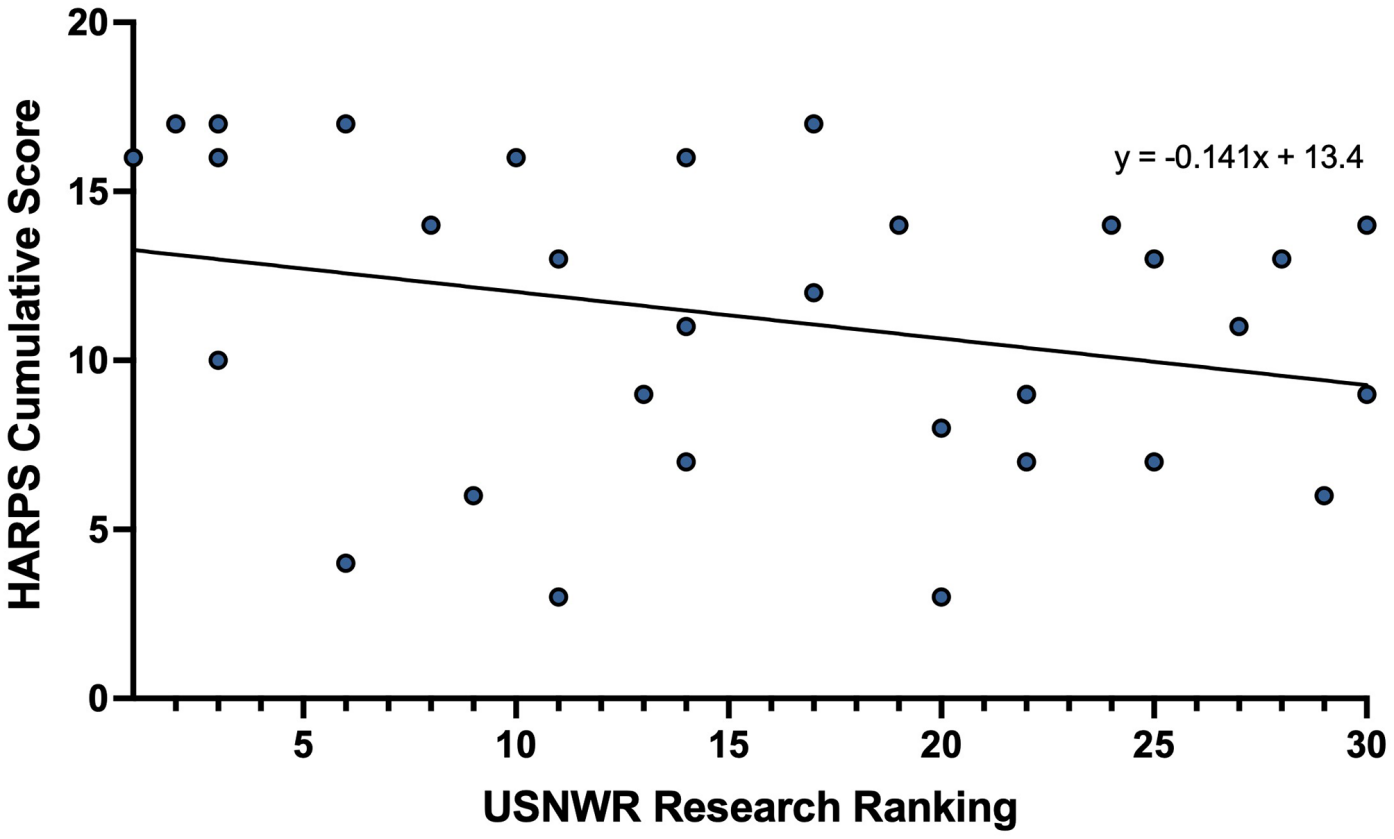
The association between cumulative HARPS score and USNWR ranking Shown is a scatter plot of the cumulative HARPS score versus USNWR ranking for the 31 schools in the study. A linear regression analysis failed to demonstrate a significant association between USNWR and the HARPS cumulative score (F [1, 29] = 2.56, p = 0.121)

**Fig. 3:**
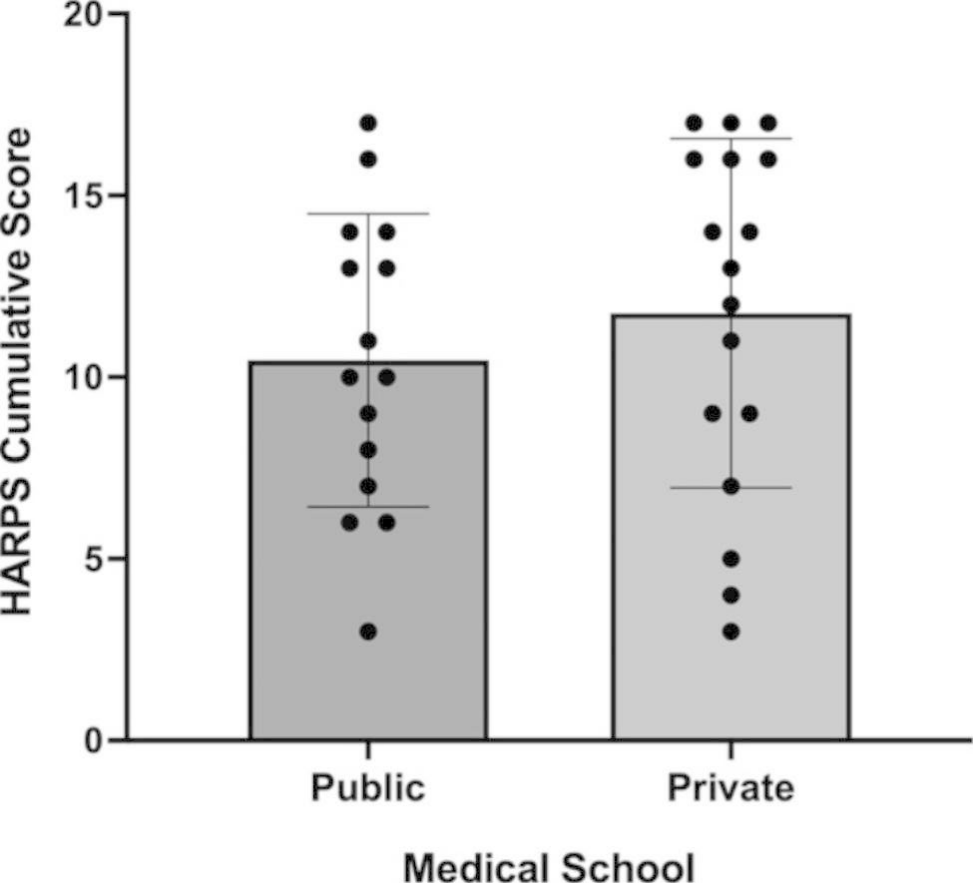
The association between cumulative HARPS score and medical status as a private versus public institution No significant differences were found between private and public institutions in their cumulative HARPS score (p = 0.626, Mann-Whitney U-Test, two-tailed)

**Fig. 4:**
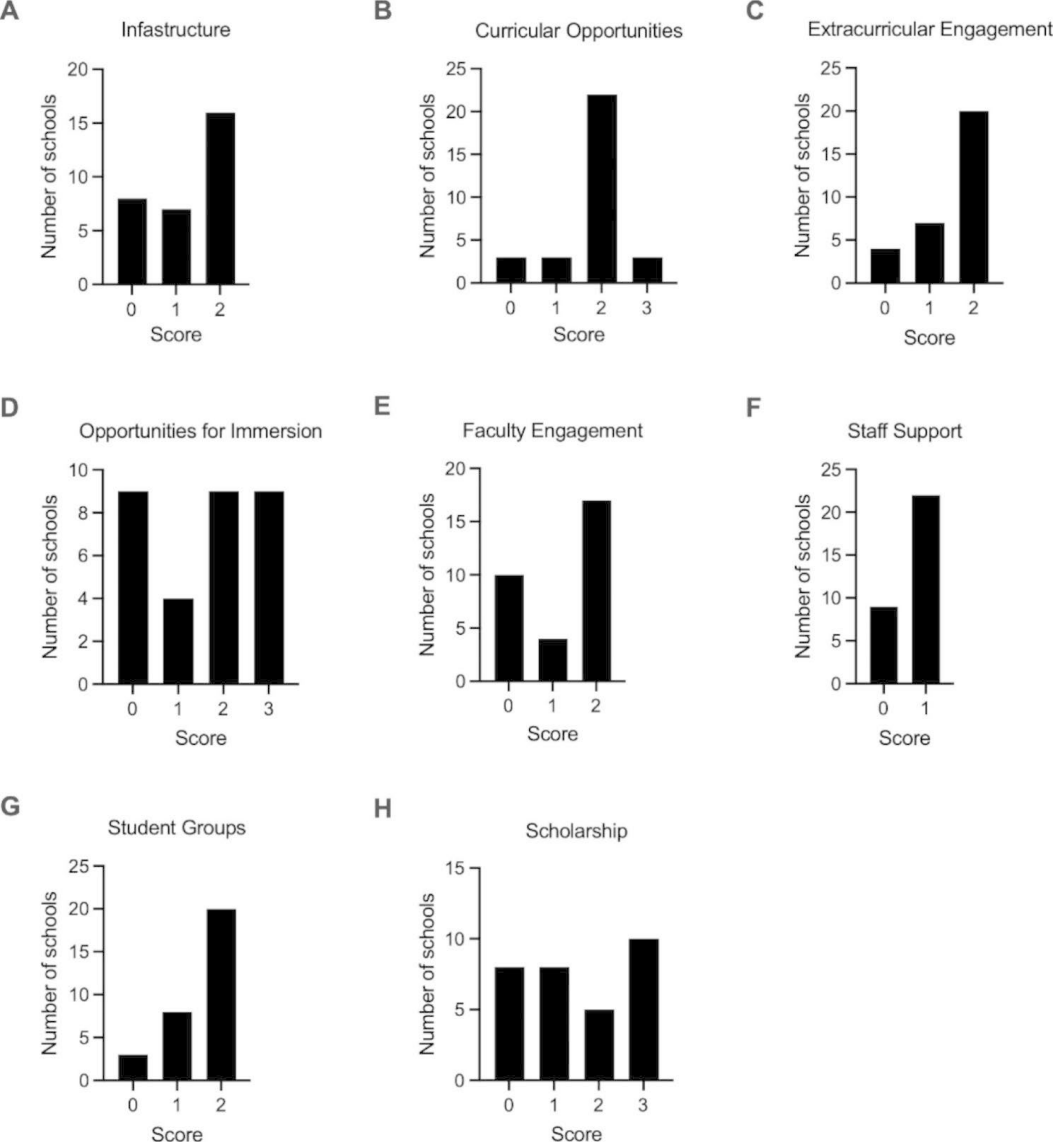
Frequency distributions of the score for each of the HARPS sub-domain Shown are the frequency distributions of the scores for each of the eight HARPS sub-domains as defined in Table 1 and derived in Table 2. Means, median and standard deviations for each sub-domain are presented in Table 3

Overall, our data reveal a significant presence of the medical humanities throughout UME as suggested by the high median cumulative HARPS score of 12 for the 31 medical schools studied (Table 3). In line with this, our data demonstrate that medical humanities no longer appear to be a niche discipline confined to a minority of extraordinarily committed or well-funded institutions. This is evidenced by our findings that the cumulative HARPS score was not associated with USNWR ranking or an institution’s public versus private status (Figs. 2 and 3) and numerous exemplars of the various domains were identified throughout the list of schools. Additionally, more than half of the institutions surveyed had a humanities-focused center or division (Fig. 4A), with more than 70% of the schools having significant (more than five) faculty engaged in the medical humanities (Fig. 4E) as well as staff support (Fig. 4F). That said, the integration of the arts and humanities does vary widely (Fig. 1), with six schools receiving a HARPS score ≤ 6, indicating that some institutions lag significantly behind their peers in their efforts to build environments conducive for the medical humanities.

Our data identified two areas of strength with respect to the presence of medical humanities in UME. The opportunity for extracurricular engagement was strong with more than two-thirds of schools offering 10 or more events annually related to arts and humanities (Fig. 4C), while nearly 70% of the institutions surveyed had more than five student organizations focused on the medical humanities or related creative disciplines (Fig. 4G). Student groups are especially important as they provide outlets for interested students, sponsor relevant programming, are sources of innovative programing and advocate for institutional change. They also likely fill the void at schools where the institutional engagement in the medical humanities is weak. That said, institutional integration of the arts and humanities is not sustainable by student energy and passion alone. Annual turnovers in student group leadership and interest in or capacity for engaging in curricular efforts will likely vary from year to year, inevitably hindering longitudinal integration.

In addition to these areas of strength, our data suggest areas for focus and growth, including curricular opportunities (Fig. 4B) and opportunities for immersion (Fig. 4D). While the overwhelming majority (22/31) of institutions offered non-credit medical humanities electives for their students, only three medical schools required explicit instruction in the arts and humanities. Further, of the eight highest performing institutions (HARPS score ≥ 16), only two had required curricular components. These data suggest that there are significant opportunities to integrate the arts and humanities into the required core curricula in UME. Considering the substantial and growing evidence of the value of the arts and humanities in fostering clinical as well as affective, cognitive, and relational skills, the urgency for curricular integration becomes even more compelling [16, 18, 23, 24]. Additionally, in only 29% of the institutions surveyed were there formal pathways for students to have a comprehensive (research and classwork), immersive experience in the medical humanities, with the same proportion offering either a limited research or classwork-only tract. This finding, together with the lack of required coursework, means that many medical students, assuming they have the interest and are motivated to seek it out, may only experience the medical humanities through student-initiated programing, occasional institutional events, and/or a limited number of elective courses.

With respect to scholarship in the medical humanities, research outputs varied widely (Fig. 4H). Research scores were significantly higher near the top of USNWR rankings (data not shown), consistent with the weighting of the USNWR rankings toward research funding. Active scholars and scholarship in the arts and humanities provides content experts for course/program development, instruction and evaluation, mentors for students, and opportunities for student research, and so contributes to the development of a strong institutional presence for the medical humanities.

### Strengths and limitations

An important contribution of this work to the literature is the development of the HARPS as a tool for the structured evaluation of the integration of the arts and humanities within UME. While further validation and refinement are ongoing to determine its range of use within and outside of UME, we believe this instrument may provide a rubric for evaluating the presence, scope, and depth of the medical humanities at a medical school and so eventually enable benchmarks for excellence and milestones of growth. Several limitations, however, should be noted. First, the medical schools reviewed were derived from the USNWR ranking. Although our study involved 20% of US allopathic medical schools, with a mix of both private and public medical schools, as well as stand-alone medical schools not associated with a larger university, given the metrics that drive the USNWR rankings, the institutions we studied may not be fully representative of US medical schools. It is also important to note that beginning in 2023, a number of the highly ranked USNWR medical schools are no longer participating in the USNWR survey. In this regard we would highlight institutions that have been recognized for leadership and innovation in incorporating the medical humanities into their education, research, and practice (see Supplemental Material, Appendix 5) but did not meet the threshold for inclusion in this study. Examples include the University of Rochester School of Medicine and Dentistry, Sidney Kimmel Medical College at Thomas Jefferson University, and Penn State College of Medicine. Another limitation is our data source: medical school websites. This information may not be reflective of current programming or activities nor fully capture all that is pertinent to art and humanities at a particular institution. This limitation was our rationale for anonymizing the quantitative data across all institutions. It would therefore be important to confirm our findings by directly surveying these institutions in subsequent studies. In addition, our study focused on the institutional presence of the medical humanities but did not explicitly investigate the effectiveness or impact of that presence on students’ learning and professional development. Future studies should therefore also address the educational quality of the arts and humanities programing provided by an institution through strategies including direct contact with faculty and staff, student surveys and focus groups [16]. Further, as pedagogy for the medical humanities evolve and mature, there will be the need for metrics that address the educational quality and impact of this instruction as well as the credentials and expertise of the faculty providing this education, some of which might be incorporated into future iterations of the HARPS. Finally, it would be of particular interest to study the long-term impact of medical students’ exposure to the humanities on their future careers as physicians, including outcomes such as professional empathy and compassion, communication skills, teamwork, professional satisfaction, and burnout.

## Conclusions

This report documents a significant presence of the medical humanities in UME in the United States, with more than 50% of studied institutions having a humanities-focused center or division as well as significant faculty and staff engagement in this area. While students are able to readily engage the arts and humanities through paracurricular events and through medical humanities student groups, the integration of the arts and humanities into the required core UME curricula and into comprehensive, immersive pathways for engaging the medical humanities are much less common. Therefore, in line with the recommendations of the FRAHME initiative,[16] an important next step in extending the medical humanities in UME should involve the integration of effective competency-based teaching and learning of the arts and humanities into longitudinal, immersive experiences and core curricula.

### Electronic supplementary material

Below is the link to the electronic supplementary material.

**Table S1**. HARPS scores by US News Ranking. **Table S2**. HARPS scores for public and private medical schools. **Figure S1**. HARPS scores by private versus public status. Box plots comparing public and private institutions across the eight HARPS domains. Detailed breakdown found in Table S2.

## Data Availability

The datasets used and/or analyzed during the current study available from the corresponding author on reasonable request.
