# Supplementary material for: The medical humanities at United States medical schools: a mixed method analysis of publicly assessable information on 31 schools
Source: BMC Med Educ. 2023 Sep 1;23:620. doi: 10.1186/s12909-023-04564-y (PMC10472551; doi:10.1186/s12909-023-04564-y)
Supplement: Supplementary file 1 — Table S1. HARPS scores by US News Ranking. Table S2. HARPS scores for public and private medical schools. Figure S1. HARPS scores by private versus public status. Box plots comparing public and private institutions across the eight HARPS domains. Detailed breakdown found in Table S2. [file 12909_2023_4564_MOESM1_ESM.docx]

**Supporting Information**

**The Medical Humanities at United States Medical Schools: A Mixed Method Analysis of Publicly Assessable Information**

Joshua Anil, Phoebe Cunningham, C. Jessica Dine MD MSHP

Amanda Swain, MD, Horace M. DeLisser, MD

Table S1 (p. 2)

Table S2 (p. 3)

Figure S1 (p. 4)

Appendix 1 (p. 5)

Appendix 2 (pp. 6-8)

Appendix 3 (pp. 9-11)

Appendix 4 (pp. 12, 13)

Appendix 5 (p. 14)

**Table S1. HARPS scores by US News Ranking**

|  | | | | | | | | | |
| --- | --- | --- | --- | --- | --- | --- | --- | --- | --- |
|  | **Infrastructure** | **Curricular Opportunities** | **Extracurricular Programming** | **Opportunities for Immersion** | **Faculty Engagement** | **Staff Support** | **Student Groups** | **Scholarship** | **Cumulative**  **Score** |
| Top 5 |  |  |  |  |  |  |  |  |  |
| Avg. | 1.80 | 2.20 | 1.60 | 2.00 | 2.00 | 1.00 | 1.80 | 2.80 | 15.20 |
| Median | 2.00 | 2.00 | 2.00 | 2.00 | 2.00 | 1.00 | 2.00 | 3.00 | 16.00 |
| SD | 0.40 | 0.40 | 0.80 | 0.63 | 0.00 | 0.00 | 0.40 | 0.40 | 2.64 |
|  |  |  |  |  |  |  |  |  |  |
| Top 10 |  |  |  |  |  |  |  |  |  |
| Avg. | 1.60 | 2.20 | 1.40 | 1.70 | 1.60 | 0.90 | 1.60 | 2.30 | 13.30 |
| Median | 2.00 | 2.00 | 2.00 | 2.00 | 2.00 | 1.00 | 2.00 | 3.00 | 16.00 |
| SD | 0.66 | 0.40 | 0.80 | 1.10 | 0.80 | 0.30 | 0.66 | 1.19 | 4.63 |
|  |  |  |  |  |  |  |  |  |  |
| Top 15 |  |  |  |  |  |  |  |  |  |
| Avg. | 1.50 | 1.88 | 1.56 | 1.69 | 1.25 | 0.81 | 1.56 | 1.75 | 12.00 |
| Median | 2.00 | 2.00 | 2.00 | 2.00 | 2.00 | 1.00 | 2.00 | 2.50 | 13.50 |
| SD | 0.79 | 0.86 | 0.70 | 1.16 | 0.90 | 0.39 | 0.61 | 1.35 | 4.77 |
|  |  |  |  |  |  |  |  |  |  |
| Bottom 15 |  |  |  |  |  |  |  |  |  |
| Avg. | 1.00 | 1.73 | 1.47 | 1.47 | 1.20 | 0.60 | 1.67 | 1.33 | 10.47 |
| Median | 1.00 | 2.00 | 2.00 | 2.00 | 2.00 | 1.00 | 2.00 | 1.00 | 11.00 |
| SD | 0.82 | 0.57 | 0.72 | 1.20 | 0.91 | 0.49 | 0.60 | 0.94 | 3.70 |
|  |  |  |  |  |  |  |  |  |  |
| All |  |  |  |  |  |  |  |  |  |
| Avg. | 1.26 | 1.81 | 1.52 | 1.58 | 1.23 | 0.71 | 1.61 | 1.55 | 11.26 |
| Median | 2.00 | 2.00 | 2.00 | 2.00 | 2.00 | 1.00 | 2.00 | 1.00 | 12.00 |
| SD | 0.84 | 0.74 | 0.71 | 1.19 | 0.91 | 0.45 | 0.61 | 1.19 | 4.35 |

**Table S2. HARPS scores for public and private medical schools**

|  | | | | | | | | | | | | | | |  |  |
| --- | --- | --- | --- | --- | --- | --- | --- | --- | --- | --- | --- | --- | --- | --- | --- | --- |
|  | **Infrastructure** | **Curricular Opportunities** | **Extracurricular Engagement** | **Opportunities for Immersion** | **Faculty Engagement** | | **Staff Support** | | **Student Groups** | | | **Scholarship** | | **Sum** | | |
| Public Avg. | 1.08 | 1.92 | 1.5 | 1.58 | 1.25 | | 0.75 | | 1.42 | | | 1.42 | | 10.92 | | |
| Public Median | 1 | 2 | 2 | 2 | 2 | | 1 | | 1.5 | | | 1 | | 10.5 | | |
| Public SD | 0.76 | 0.28 | 0.76 | 1.26 | 0.92 | | 0.43 | | 0.64 | | | 1.11 | | 3.68 | | |
|  | | | | | | | | | | | | | | |  |  |
| Private Avg | 1.37 | 1.74 | 1.53 | 1.58 | | 1.21 | | 0.68 | | 1.74 | 1.63 | | 11.47 | | |  |
| Private Median | 2 | 2 | 2 | 2 | | 2 | | 1 | | 2 | 2 | | 13 | | |  |
| Private SD | 0.87 | 0.91 | 0.68 | 1.14 | | 0.89 | | 0.46 | | 0.55 | 1.22 | | 4.72 | | |  |

**Figure S1. HARPS scores by private versus public status**

Box plots comparing public and private institutions across the eight HARPS domains. Detailed breakdown found in Table S2.

**
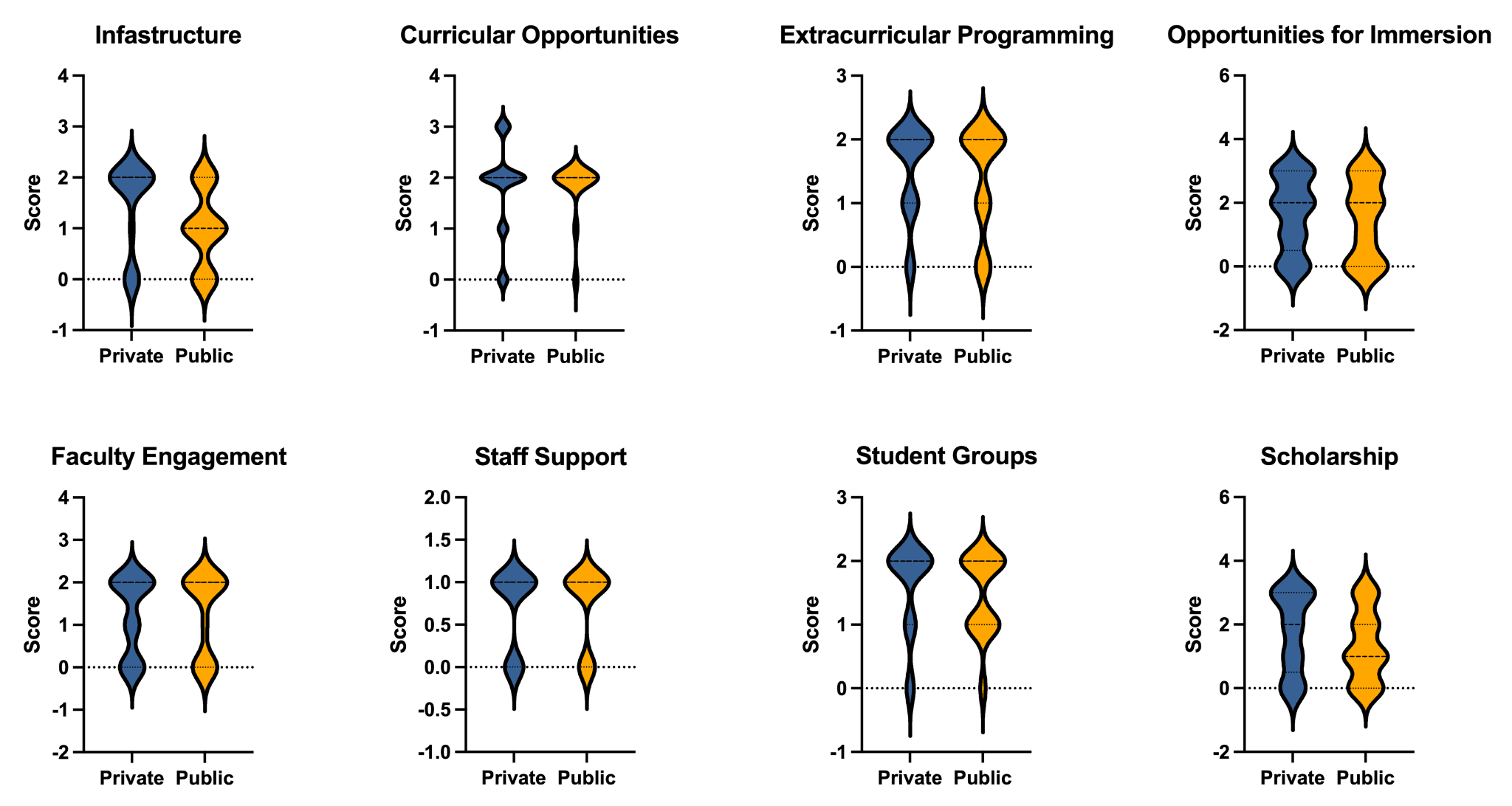
**

**APPENDIX 1**

**List of Evaluated Schools based on USWNR Research Ranking**

Schools listed in 2022-2023 USNWR Ranking Order (tied schools placed in alphabetical order)

1. Harvard University
2. New York University (Grossman)
3. Columbia University **(tie for 3)**
4. Johns Hopkins University **(tie for 3)**
5. University of California -- San Francisco **(tie for 3)**
6. Duke University **(tie for 6)**
7. University of Pennsylvania (Perelman) **(tie for 6)**
8. Stanford University
9. University of Washington
10. Yale University
11. Icahn School of Medicine at Mount Sinai **(tie for 11)**
12. Washington University in St. Louis **(tie for 11)**
13. Vanderbilt University
14. Cornell University (Weill) **(tie for 14)**
15. Mayo Clinic School of Medicine (Alix) **(tie for 14)**
16. University of Pittsburgh **(tie for 14)**
17. Northwestern University (Feinberg) **(tie for 17)**
18. University of Michigan -- Ann Arbor **(tie for 17)**
19. University of California -- Los Angeles
20. University of California -- San Diego **(tie for 20)**
21. University of Chicago (Pritzker) **(tie for 20)**
22. Baylor College of Medicine **(tie for 22)**
23. Emory University **(tie for 22)**
24. Case Western Reserve University
25. University of North Carolina -- Chapel Hill **(tie for 25)**
26. University of Texas Southwestern Medical Center **(tie for 25)**
27. University of Colorado
28. University of Southern California (Keck)
29. University of Maryland
30. Ohio State University **(tie for 30)**
31. University of Virginia **(tie for 30)**

**APPENDIX 2**

**Development of the Humanities and Arts Programming Scale (HARPS)**

- - - 1. **Context**

No metric existed to evaluate medical humanities offerings within UME. While scoping reviews had been performed, prior research focused on assessing the content domain areas of programming and classifying opportunities on pedagogical function, such as Social Advocacy, Personal Insight, Perspective Taking, and Mastering Skills. Other scholarship has used student feedback forms as a metric to evaluate the quality and efficacy of medical humanities programming. However, no ecological approaches were taken in the literature. Arts and Humanities inclusion within UME was assessed in a vacuum, without consideration of the supporting infrastructure and encompassing foundations that promote engagement. In order to assess the ecology of medical humanities in UME, the Humanities and Arts Programming Scale (HARPS) was developed.

- - - 1. **Personnel**

The core research team contained several experts with extensive experience in medical humanities, as well as medical education. Research team members were responsible for organizing humanities development at their home institution, while also serving in national capacities on Arts & Humanities within UME. Additional experts in UME assessment and evaluation were also consulted throughout the scale development process.

- - - 1. **Development**

Development of the scale began with a review of relevant literature, including the AAMC FRAHME report. This review led to the generation of a select number of themes critical to creating a humanistic ecology. Themes included concepts such as individual exploration, intentional mentorship, formal education, administrative support, and community-building. Once themes were identified, a variety of assessable characteristics were generated among all members of the project team. These characteristics were assessed for feasibility, efficacy, and reproducibility. Groups of characteristics were selected and tested on randomly selected institutions to evaluate the usefulness and collectability of each metric. Results from each creation test were discussed with the entire research team, and refinements were made to improve the utility of the scale in future tests. Research team members with expertise in UME and medical humanities assisted in ensuring content validity of the developing instrument. After several rounds of testing, the researchers determined the following 8 domains provided a holistic evaluation of the critical themes: Infrastructure, Curricular Opportunities, Extracurricular Engagement, Opportunities for Immersion, Faculty Engagement, Staff Support, Student Groups and Scholarship. A brief justification for each of the criteria is provided

- ***Infrastructure.*** The presence of a dedicated center, institute, or department for the medical humanities connotes a substantial level of institutional investment, and grants significant stability to the programming and other offerings present. It is difficult to create an environment for medical students to encounter the medical humanities without providing a logistical hub or institutional home for related content. This domain served as a partial surrogate for the theme of administrative support, along with the domains of faculty engagement and staff support.
- ***Curricular Opportunities***. Curricular incorporation of the medical humanities is something that has been assessed many times in the literature, and is the primary existing method for evaluation of medical humanities education. As such, we included it as one of our core domains.
- ***Extracurricular Engagement***. To properly assess the humanistic ecology of an institution required assessment of opportunities for engagement and education outside of standard curricular and mentored tracks. Extracurricular programming in the forms of talks, symposiums, community engagement, and much more provide reinforcing experiences to students while also highlighting concepts and themes that are more difficult to convey in a standardized classroom setting. Extracurricular engagement also allows for student exploration of specific fields within the medical humanities, allowing for individualized exploration, and aiding in the creation of a multidisciplinary space that promotes longitudinal critical engagement.
- ***Opportunities for Immersion***. This domain emerged in response to the themes of individual exploration and intentional mentorship. While the HARPS scale does not assess the content or complexity of medical humanities offerings, we wanted to ensure that the measure relayed some level of depth information. Immersion opportunities such as Scholarly Pursuits/Investigations, Certificates, or Areas of Concentration have continued to become more and more frequent across US Medical Schools. Assessing the inclusion of medical humanities within formal longitudinal research, coursework, or mentorship tracks is an indicator of parity between the medical humanities and other traditional disciplines, while also serving as a marker of program depth and investment into the humanities.
- ***Faculty Engagement.*** Faculty Engagement was viewed as critical to the development and mentorship of medical students encountering the medical humanities. Just as instruction is paramount in traditional aspects of UME, the research team felt that quality engagement from qualified preceptors and faculty members was vital to developing sustainable humanities ecology. This domain also served as a partial surrogate for the theme of administrative support community-building, and structured mentorship.
- ***Staff Support.*** The level of dedicated staffing support can dramatically impact the activity and accessibility of programming. Staff presence removes burden from both faculty balancing clinical, research, and administrative responsibilities and students who have neither the time, expertise, or administrative memory to effectively develop sustainable multi-faceted programming.
- ***Student Groups.*** The formation of communities was reinforced by our literature search, which frequently revealed scattered sets of interested individuals or isolated programs at institutions, but has more limited information regarding long-term sustained interest in humanistic endeavors. Student groups provide a partial surrogate for community-building by providing a space for interested students to engage with not only the medical humanities, but also other students passionate about these creative disciplines. As such, the level of student group presence may be indicative of the sustainability and activity of an institution’s medical humanities opportunities.
- ***Scholarship.*** As research is a vital part of the academic medical school training pathway, and scholarly inquiry is becoming more and more encouraged regardless of future career trajectories, we felt it was important to evaluate the presence of original humanities investigation across institutions. Scholarship serves as a surrogate for several factors. It demonstrates that there are interested individuals with some level of expertise. It highlights that humanistically-driven research is welcomed and accepted to a similar level to basic and clinical research. It points to the opportunity for UME students to become involved in meaningful projects that can serve as catalysts for individual exploration, community-building, and intentional mentorship.

Once the domain areas were determined, classifications were created through discussion with the full research team. Classifications were chosen to provide clear demarcations between levels of performance in various domains. Various classification schemes were developed for each domain and trialed on randomly selected medical schools to determine accuracy and validity. All results from selected schools were reviewed with the research team to determine classification adjustments and develop final classifications.

**APPENDIX 3**

**Curricular Integration**

Coursework in the medical humanities came in a variety of formats and touched on a multitude of disciplines. We provide an overview of coursework timing and content while providing a selection of specific courses obtained during our review, in order to provide a better understanding of the range and structure of successfully implemented coursework in the medical humanities.

**Timing of courses/curricular opportunities**

Humanities courses/curricular opportunities fall in 1 of 3 periods:

- Preclinical (Traditionally the 1st and 2nd years of medical school)
- Core Clinical Year
- Non-Clinical (The 3rd or 4th year of medical education, including protected time for research and professional development)

**Levels of participation**

- Courses have varying levels of participation:
- **Level 1:** Universally Required - All students at the institution take the same course in the same general time frame
- **Level 2:** Required Selectives - All students at the institution must receive some set of medical humanities instruction, but each student is able to tailor their education to their specific interests
- **Level 3:** Elective (Credit) - Students must opt-in to taking an elective class that counts towards completion of degree requirements.
- **Level 4:** Elective (No Credit) - Students must opt-in to taking a medical humanities class that does not go towards completion of degree requirements. Transcript notation may or may not be provided.
- Single institutions may have courses that fall under different levels of participation. For illustration, below is a sampling of Yale Medical School Medical Humanities Curriculum: (<https://medicine.yale.edu/humanitiesinmedicine/curriculum/>)
- Level 1
- Reflective Writing Workshops **[Clinical Year]**
  - Integrated into Core Clinical Year curriculum, occur 4 times a year
  - Led by senior students and provides structured environment for individual and group reflection
- Making the Invisible Visible: Exploring Bias Through Art **[Pre-Clinical]**
  - Mandatory guided art tour and reflection session that occurs during first preclinical course
- Level 3 or 4
- Non Clinical
- Seminar in Healer’s Arts

The Healer’s Art course utilizes principles of adult education, contemplative studies, humanistic and transpersonal psychology, cognitive psychology, formation education, creative arts and storytelling to present and explore human dimensions of medicine rarely discussed in medical training.

- Seminar in Medical (Mal)Practices Under the Nazi Regime
- Seminar in Life Worth Living

This is a course of applied philosophy, where we address questions of meaning in our profession and reflect upon our own practice.

**3. The range of content**

To highlight the diverse range of content areas, provided below is a selection of elective names from several institutions. Note: these are not comprehensive lists, and redundant electives across institutions may not be listed (i.e. Healer’s Art will not be listed for every school that offers it). Additionally, elective offerings frequently change. Courses listed below are a snapshot of the opportunities available at data collection. Please refer to links and references for the most up-to-date information.

- Vagelos College of Physicians and Surgeons at Columbia University - Level 2 Courses **[Pre-Clinical]**
- Poetry: Close Readings and Craft
- The City of the Hospital: The Medical Student as Writer
- Opinion Writing as Resistance
- Movement as Story: an Exploration of Dance and the Spectrum of Physical Narrative
- The Art of Paying Attention
- Works of Art as a Way of Knowing
- Comic-book Storytelling Workshop
- Dreams and the Unconscious
- Photography and Visual Storytelling
- Life Stories of Anatomic Donors: An Obituary Writing Workshop
- Making Meaning: Using Emotion to Foster Relationships Essential to the Practice of Medicine
- Short Fiction Workshop
- Reporting Live from NYP: Journalistic Writing for Doctors
- Race Sounds: The Art of Listening in African American Literature and Music Attending to Movies: Affect and Insight
- University of Virginia School of Medicine - Level 3 Courses **[Post-Clinical]**

(<https://med.virginia.edu/biomedical-ethics/education/eleectives-for-fourth-year-medical-students/>)

- Literature and Medicine
- Images of Medicine in Film, Literature, and Visual Arts
- Mindful Practice/Mindful Life
- Suffering, Medicine, and Faith
- History of Medicine
- Public Health in Fiction and Film
- Ethics, Society, and Human Biology
- The Calls of Medicine
- Independent Research in Humanities
  - - - University of Texas Southwestern (UTSW): Level 4 Courses **[No specific Timepoint]** (<https://utsouthwestern.smartcatalogiq.com/en/2022-2023/Medical-School/Courses>)
- ENRH 130 MEDICINE & CINEMA
- ENRH 142 HUMANISM IN MEDICINE
- ENRH 145 RELIGION & MEDICINE
- ENRH 148 CULINARY MEDICINE
- ENRH 150 MEDICINE AND ART
- ENRH 152 MEDICINE & THE END OF LIFE
- FAM 2006W THE ART OF NARRATIVE MEDICINE
- GEN 2102 LAW MEDICINE & LIT
- LIB 2001 EXPLORE MEDICAL HUMANITIES
- MED 2119 BUILDING A FOOD FOUNDATION
- OBG 2114W MED IN CINEMA: WOMEN'S ISSUES
- PSY 2116W MINDFULNESS FOR MED STUDENTS
- Stanford University School of Medicine

(<https://med.stanford.edu/medicineandthemuse/Education/CoursesofInterest.html>): Level 3 or 4 **[No Specific Time Point]**

- INDE 273: Medical Improvisation
- EMED 228: Virtual Reality Storytelling
- EMED 205: Film and Television Emergencies: Grasp Emergency Care through Pop Culture
- MED 201: Internal Medicine: Body as Text
- MED 234: Literature and Global Health
- SOMGEN 216: Medical Etymology
- SOMGEN 221: Media, Medicine & (Mis)information
- University of California at San Francisco School of Medicine

UCSF (<https://coursecatalog.ucsf.edu/medicine>): Level 3 or 4 **[No Specific Time point]**

- 130.21 CIEx - Medical Student as Writer
- 140.22 Narrative Medicine and Podcasting with The Nocturnists
- 141.16 Social Justice and Medicine in the East Bay
- 170.48 A Student-Curated Health Humanities Event Series
- 170.01 An Introduction to Medical Anthropology
- 140.60 Expanding WikiProject Medicine (6 units) Fall, Winter, Spring, Summer

**APPENDIX 4**

**Detailed Student Group Information**

- - - 1. **Overview**

Student groups related to the medical humanities cover a breadth of disciplines and demonstrated a strong domain across medical schools. Driven by student passions and time, many student groups have the capacity to explore more specific areas such as knitting, advocacy performances, and Latin dance. The below section described the common themes seen throughout student groups with specific examples from three schools.

- - - 1. **Common Themes**
- Literary Arts
  - Book Clubs centered on religion, advocacy, history etc.
  - Narrative Medicine Interest Groups or Societies
  - Literary Magazines
  - Storytelling Projects
- Music
  - A Capella
  - Medical School Orchestras
  - Choirs (classical, rock, jazz)
  - Bedside Concerts
  - Music and Medicine Interest Groups
- Performance and Film
  - Film and Television Clubs
  - Dance Groups (Latin dance, ballet, general)
  - Comedy and Improv Groups
- Visual Arts
  - Painting Clubs
  - Knitting Groups
  - Art and Medicine Groups
  - Crafting Groups
- Other
  - History of Medicine Groups
  - Bioethics Groups
  - Gardening Groups
  - Cooking Arts
- Therapeutic Arts

**3. Examples from representative schools**

- University of Maryland School of Medicine - Student Groups

<https://medscope.umaryland.edu/StudentGroups>

- Creative HeArts
- Hippocratic Notes
- UMSOM Film and Literature Society
- Medical Ethics Interest Group
- History of Medicine Interest Group
- Gold Humanism Honor Society
- Ohio State School of Medicine - Student Groups

<https://medicine.osu.edu/student-resources/student-organizations/humanism-in-medicine>

- College of Medicine Orchestra
- Cultural Cooking
- Dance in Medicine
- Medical Student Improv Group
- On Call Creations
- Photography Group
- Theater/Film Arts
- UltraSound A Cappella
- Vagina Monologues Student Activists
- Writer’s Group/Ether Arts
- Cornell Weill School of Medicine - Student Groups

<https://medicaleducation.weill.cornell.edu/student-life/student-groups-community-service>

- AneuRhythms - A Capella Group
- Ascensus, Journal of Humanities
- Ballet Interest Group
- ChefED
- Cornell Capoeira
- Cornell Crafts Club
- Gardening and Conspiracies
- Happy Feet Holistic Healing at WCMC
- Music and Medicine Initiative
- Nature Rx WCM
- WCMC Art Club
- Conversations in Medical Ethics

**APPENDIX 5**

**Evaluation of Additional Institutions**

We realize that the restriction of our evaluation to schools listed in the USNWR Research ranking is imperfect. We therefore wanted to briefly highlight three other institutions beyond the 31 schools that were the focus of this study. The three selected schools - the Sidney Kimmel Medical College of Thomas Jefferson University, the University of Rochester School of Medicine and Dentistry, and Pennsylvania State College of Medicine - span the gamut in size, private versus public status and ranking. Yet they have in common a strong dedication to incorporating the medical humanities into their education, research, and practice.

**The Sidney Kimmel Medical College of Thomas Jefferson University**

Jefferson’s Humanities and Health Center serves their entire health-focused institution, and puts on 50+ annual events to give students and faculty a chance to explore and grow their understanding of the medical humanities. Additionally, Jefferson requires its medical students to complete 2 medical humanities selectives, with offerings spanning from music and art, to history, film, and writing. Coupled with a Scholarly Inquiry track in the humanities that allows students to perform innovative research, a certificate program that rewards students for engagement in the Center’s numerous events, and 10+ humanities student groups, Jefferson requires baseline engagement with the medical humanities, while creating opportunities for students to dive deeper if interested.

**The University of Rochester School of Medicine and Dentistry**

The University of Rochester, as one of the earliest institutions to create a medical humanities division, is similarly invested in humanistic medical education. They, like Jefferson, also require all medical students to participate in two medical humanities selectives in Phase 1 and 2 of their curricula, while offering summer and year-out fellowships in the health humanities for interested students. Their enrichment pathway in humanities combines both classwork and scholarly production to create meaningful opportunities for longitudinal learning. Finally, their center for health humanities complements these curricular efforts by hosting regular lunch talks, supporting 20+ faculty, and serving as an administrative home for health humanities-related efforts at the institution.

**Pennsylvania State College of Medicine**

Finally, the Pennsylvania State College of Medicine provides another excellent example of how publicly funded schools can have strong medical humanities cultures. The first institution in the country to create a medical humanities department, their commitment to the medical humanities is present in each phase of their undergraduate medical curriculum. In Phase 1, students have humanities coursework every Tuesday morning, while during Phase 2 of clerkships, they participate in backstory rounds to continue longitudinal humanities development. Finally, Phase 3 of the curriculum requires a month-long humanities selective. In addition to their curricular strengths, PSU’s college of medicine Department of Humanities has a robust research program, with the department’s 11 faculty producing dozens of cited reviews and studies over the past 5 years.
